# Association of congenital heart disease and neurodevelopmental disorders: an observational and Mendelian randomization study

**DOI:** 10.1186/s13052-024-01610-3

**Published:** 2024-04-08

**Authors:** Zhi-yuan Liu, Qiong-qiong Wang, Xian-yong Pang, Xiao-bi Huang, Gui-ming Yang, Sheng Zhao

**Affiliations:** 1 Department of Cardiology, Anhui Provincial Children’s Hospital, Hefei, Anhui China; 2 The Fifth School of Clinical Medicine, Anhui Medical University, Hefei, Anhui China

**Keywords:** Congenital heart disease, Neurodevelopmental disorders, Mendelian randomization, Propensity score matching

## Abstract

**Background:**

This study aims to thoroughly study the connection between congenital heart disease (CHD) and neurodevelopmental disorders (NDDs) through observational and Mendelian randomization (MR) designs.

**Methods:**

This observational study uses data from the National Survey of Children’s Health (2020–2021). Multivariable logistic regression and propensity score matching (PSM) were performed to analyze the association. PSM was used to minimize bias for covariates such as age, race, gender, maternal age, birth weight, concussion or brain injury, preterm birth, cerebral palsy, Down syndrome, and other inherited conditions. In MR analyses, inverse variance-weighted measures, weighted median, and MR-Egger were employed to calculate causal effects.

**Results:**

A total of 85,314 children aged 0–17 were analyzed in this study. In regression analysis, CHD (*p* = 0.04), the current heart condition (*p* = 0.03), and the severity of current heart condition (*p* < 0.05) had a suggestive association with speech or language disorders. The severity of current heart condition (*p* = 0.08) has a potential statistically significant association with attention deficit hyperactivity disorder(ADHD). In PSM samples, ADHD(*p* = 0.003), intellectual disability(*p* = 0.012), and speech or language disorders(*p* < 0.001) were all significantly associated with CHD. The severity of current heart condition (*p* < 0.001) also had a significant association with autism. MR analysis did not find causality between genetically proxied congenital cardiac malformations and the risk of NDDs.

**Conclusions:**

Our study shows that children with CHD have an increased risk of developing NDDs. Heart conditions currently and severity of current heart conditions were also significantly associated with these NDDs. In the future, we need to try more methods to clarify the causal relationship between CHD and NDDs.

**Supplementary Information:**

The online version contains supplementary material available at 10.1186/s13052-024-01610-3.

## Introduction

Congenital heart disease (CHD) is a common congenital structural malformation and a leading cause of infant mortality [1]. Advances in surgical and intensive care have significantly increased the survival rate of children with CHD, including those with critical CHD, to 82.5% [2]. With the prolongation of lifespan in children with CHD, the focus of current research has shifted, and neurodevelopmental disorders (NDDs), which have a major impact on their long-term quality of life have received increasing attention. In 2012, the American Heart Association issued a statement aimed at improving the assessment of and attention to neurodevelopmental outcomes in children with CHD [3]. Despite numerous studies on this topic, the exact conclusion for the neurodevelopmental outcomes of CHD is still limited to date. Even the causal association between CHD and NDDs remains unclear.

Due to the low incidence rate of CHD, the sample size of many studies is small [4, 5]. Besides, some studies only focused on children with complex CHD, such as single and left ventricular hypoplasia syndromes [6, 7]. The generalizability of conclusions from these studies may be limited or not applicable to children with more common mild or moderate cardiac malformations. Recently, Tsao [8] and Sigman et al. [9] studies with a large number of samples have found a link between CHD and NDDs, but the populations of children in these studies were limited to the medical system, which may cause selection bias. Similarly, ascertainment bias cannot be ignored when children are exposed to hospitals or treatments frequently. Additionally, many confounding factors, such as the type of cardiac malformation [10], surgical methods [11], postoperative complications [12], and gene mutations [13], can influence developmental disorders in children with CHD, which also reduces the reliability and stability of the study conclusion to varying degrees.

Therefore, we conducted a large nationwide observational study to investigate the association between CHD and NDDs. To control for confounding, we utilized propensity score matching (PSM) and regression analyses. Additionally, we performed a two-sample Mendelian randomization (MR) study, which is a statistical genetics method that draws causal inferences between exposure and outcome by utilizing genetic variants from the whole genome [14]. Since genetic variants are assigned randomly at meiosis and before disease onset, MR studies can minimize confounding biases and reverse causation [15]. To our knowledge, this is the first MR study investigating the causal relationship between CHD and NDDs. We hypothesized that there is an association and causality between CHD and NDDs in children, and congenital heart malformations increase the risk of NDDs, including autism spectrum disorder (ASD), attention deficit hyperactivity disorder, intellectual disability, and speech or language disorders. Through this comprehensive method, we hope to provide a strong scientific basis for the relationship between CHD and NDD.

## Methods

### Study design and participants

**Fig. 1:**
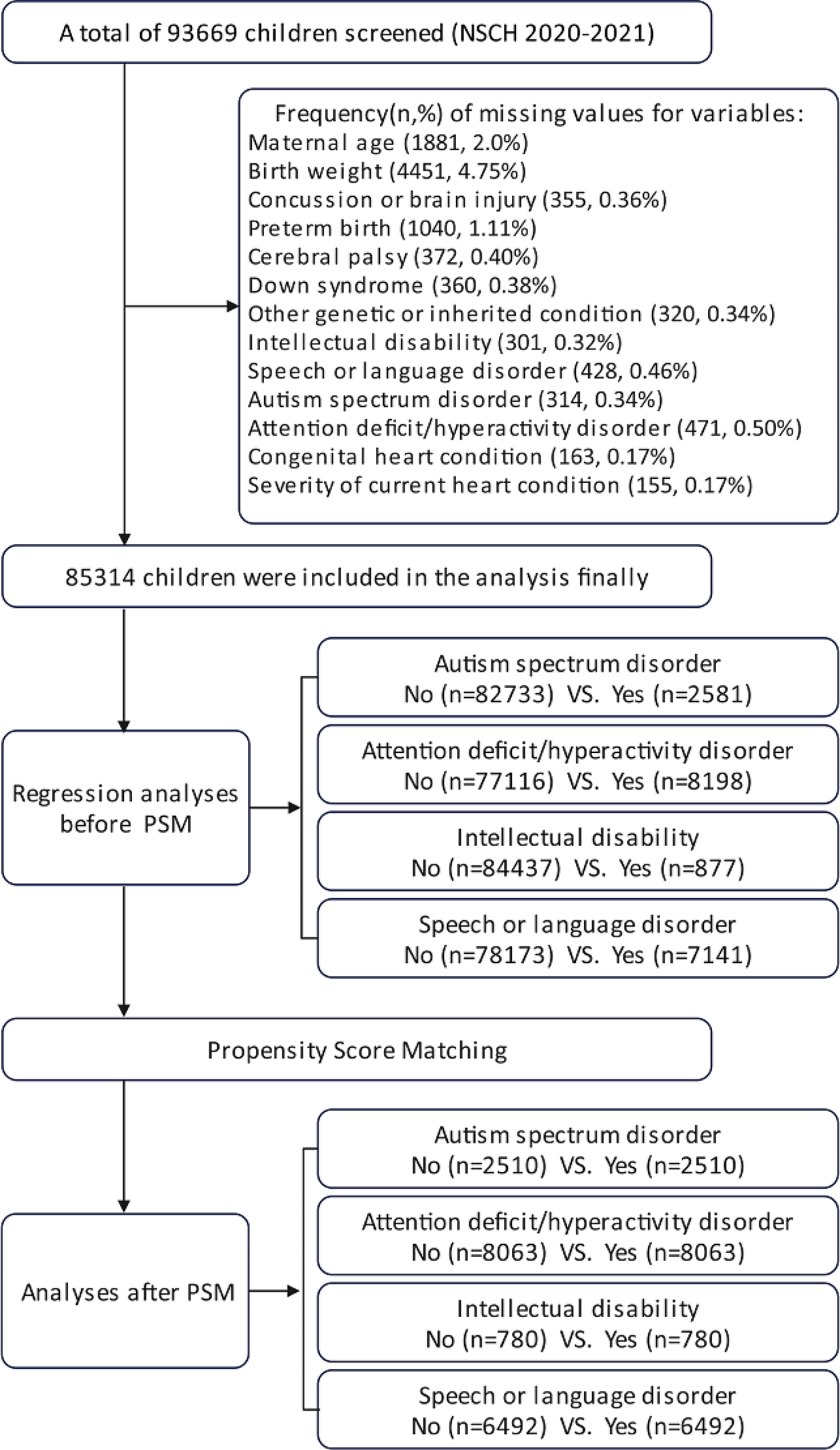
Flow chart showing the patient selection process and analysis in the observational study

The present study used cross-sectional data from the 2020–2021 National Survey of Children’s Health (NSCH), which is funded and directed by the Health Resources and Services Administration’s Maternal and Child Health Bureau [16]. The NSCH is designed to generate national and state-level data on the physical and emotional health of children in the United States aged 0–17 years. The NSCH (2020–2021) had a total of 93,669 completed surveys with an overall weighted response rate of 42.4% for 2020 (*N* = 42,777) and 40.3% for 2021 (*N* = 50,892). Participants with missing values of study variables were excluded, leaving a final analysis sample of 85,314 children. The flow of participant screening and the frequency of missing values are illustrated in Fig. 1.

### Sampling methods and procedures

In this survey, randomly selected households were contacted by mail, web, or paper (offered in both English and Spanish). In each household with children aged < 18 years old, one child was randomly chosen for participation in the survey. The survey oversampled children with special health care needs and children aged 0–5 years. Additional information regarding sampling, administration, and methodology of the NSCH can be found on the Data Resource Center website [17].

### Variables and data sources

The interest variable, congenital heart condition, was assessed based on the question, “Was this child born with the condition?” If the respondents answered “yes” to this question, the child was categorized as “yes” and “no” otherwise. In addition, respondents who answered “yes” were further asked whether the child currently has the condition, and, if so, the severity level was assessed with the question, “If yes, is it mild, moderate, or severe?” The variables of the heart condition currently and severity of current heart condition were based on these two questions, respectively. Children with current heart conditions were classified as mild, moderate, or severe based on their responses.

Autism, the outcome variable of the study, was assessed based on the question, “Has a doctor or other health care provider ever told you that this child has autism or ASD? Include diagnoses of Asperger’s disorder or pervasive developmental disorder.” ADHD, another outcome variable, was assessed based on the question, “Has a doctor or other health care provider ever told you that this child has attention deficit disorder or attention deficit/hyperactivity disorder, that is, ADD or ADHD?” Intellectual disability, another outcome variable, was assessed based on the question, “Has a doctor, other health care provider, or educator ever told you that this child has an intellectual disability (formerly known as mental retardation)?” Speech or language disorder, another outcome variable, was assessed based on the question, “Has a doctor, other health care provider, or educator ever told you that this child has a speech or other language disorder?” The child was categorized as “yes” or “no” based on the responses to all the above-mentioned questions.

Based on the risk factors for NDDs, we examined the following covariates as potential confounding variables: maternal age, birth weight (in ounces), concussion or brain injury, preterm birth, cerebral palsy, Down syndrome, and other genetic or inherited conditions. Demographic variables including age (in years), race (classified as Hispanic; White, non-Hispanic; Black, non-Hispanic; Asian, non-Hispanic; American Indian or Alaska Native Non-Hispanic; Multi-Race Non-Hispanic), and gender were also included as covariates.

### MR analysis

We conducted a two-sample MR analysis using summary-level genome-wide association study statistics data. The datasets for genetic associations with congenital heart conditions were obtained from FinnGen, which included 3,42,499 individuals of European ancestry (3,459 cases and 3,39,040 controls). Instrumental variables were selected based on the following criteria: (i) associated at the level of genome-wide significance (*p* < 5 × 10^− 6^); (ii) linkage disequilibrium r^2^ < 0.001; (iii) < 5000 KB from the index variant. A total of 14 single-nucleotide polymorphisms (SNPs) were identified and listed in Supplementary Table 1.

The outcome data for NDDs were obtained from different sources, including the Integrative Psychiatric Research-Psychiatric Genomics Consortium (18,382 cases and 27,969 controls) for ASD [18], the Psychiatric Genomics Consortium (20,183 cases and 35,191 controls) for ADHD [19], the Social Science Genetic Association Consortium (12,441 individuals) for childhood intelligence [20], and FinnGen (2,288 cases and 25,4976 controls) for speech and linguistic disorders (Supplementary Table 2). European pedigree population data were restricted to reduce the bias caused by population level.

### Statistical analysis

Observational analyses were conducted based on NSCH 2020–2021 data. Continuous data were expressed as mean ± standard deviation or median (interquartile range) (for skew distributional data). The t-test or nonparametric rank-sum test, was performed to compare the differences between the groups with and without NDDs. Categorical data are described by frequency, and the differences between groups were compared using χ2 or Fisher exact test. To estimate the association between each factor and outcome, univariate and multivariable logistic regression analysis was used, with NDDs as the outcome variable and study and confounding variables (inclusion of NDDs other than outcomes) as explanatory variables. Multivariate adjusted odds ratios (ORs), 95% confidence intervals (Cis), and p-values were calculated. To reduce the impact of confounding variables and selection bias on results, PSM was also used to match case groups to control groups. Demographic variables (age, race, and gender), confounding variables (maternal age, birth weight, concussion or brain injury, preterm birth, cerebral palsy, Down syndrome, and other genetic or inherited conditions), and NDDs (other than outcomes) were used as covariates in the PSM model, using 1:1 nearest-neighbor matching techniques with a 0.01 caliper level. In the matched samples, NDDs were the outcome variable, and heart condition-related variables (congenital heart condition, current heart condition, and its severity) were the explanatory variables. A p-value of < 0.05 indicates a statistically significant difference.

In the MR analyses, we used inverse variance-weighted measures (IVW-MR), weighted median, and MR-Egger to estimate causal effects, and results are presented as ORs with 95% CIs per standard deviation increment in exposures. The F-statistic was employed to assess the strength of the instrument, and a value of at least 10 indicates weak instrument bias [21]. Additionally, we conducted several sensitivity analyses, including MR-Egger regression, MR Pleiotropy Residual Sum and Outlier (MR-PRESSO) method, to investigate the possibility of horizontal pleiotropy (*p* < 0.05) [22, 23]. The Cochran Q test in the IVW-MR method was performed to determine heterogeneity (*p* < 0.05) [24]. We further conducted a leave-one-out analysis and made a scatter plot to visually examine possible outliers. All analyses were performed using R (version 4.2.2) and the following packages: dplyr, MatchIt, cobalt, gmodels, ggplot2, knitr, kableExtra, tableone, survey, reshape2, and TwoSampleMR.

### Research restrictions

We recognize that there are some restrictions in this study. First of all, due to the relatively low incidence of severe CHD, the sample volume of some study variables may be small. Secondly, the data sources adopted by this research may have problems with insufficient information in some aspects, such as surgical methods for CHD. In the end, although MR analysis can reduce confusion, there are still some assumptions and limitations. These restrictions will be fully discussed in the conclusion and provide inspiration for future research.

### Ethical statement

This study was approved by the Ethics Committee of Anhui children’s Hospital (approval no. EYLL-2022-020). This study involved secondary analysis using data from genome-wide association studies and the National Survey of Children’s Health (2020–2021). The participants had granted written informed consent before either study started. We confirm that all methods were performed in accordance with the ethical standards as laid down in the Declaration of Helsinki and its later amendments or comparable ethical standards.

## Results

### Observational study

A total of 85,314 children and adolescents aged 0–17 were included in the analysis, of whom 1,977 participants had CHD (2.3%), 2,581 had ASD (3.0%), 8,198 had ADHD (9.6%), 877 had intellectual disability (1%), and 7,141 had speech or language disorders (8.3%). The baseline characteristics of children with and without NDDs are presented in Table 1. The Kruskal-Wallis rank sum test and χ2 test indicated significant differences in covariates between groups with and without NDDs (*p* < 0.001). Then we conducted a multivariable logistic regression analysis to explore the association between congenital heart conditions and the risk of NDDs (Table 2 and Supplementary Table 3). For ADHD, the severity of current heart condition (OR, 2.4; 95% CI, 1.4–3.4; *p* = 0.08) has a potential statistically significant association with it. For speech or language disorders, regression analysis showed that congenital heart disease (OR, 1.61; 95% CI, 1.14–2.08; *p* = 0.04), the current heart condition (OR, 2.45; 95% CI, 1.65–3.25; *p* = 0.03), and the severity of current heart condition (*p* = 0.01 for mild, *p* = 0.02 for moderate) had a suggestive association with it. However, these variables were not associated with ASD or intellectual disability (*p* > 0.05) in multivariable regression analyses. Notably, the association between current severe heart conditions and NDDs was not available due to the small number of cases in groups.

**Table 1:** Baseline characteristics of children according to the presence of neurodevelopmental disorders

Characteristics	Autism spectrum disorder			Attention deficit/hyperactivity disorder			Intellectual disability			Speech or language disorder		
	No (*N* = 82,733)	Yes (*N* = 2581)	*p* value	No (*N* = 77,116)	Yes (*N* = 8198)	*p* value	No (*N* = 84,437)	Ye (*N* = 877)	*p* value	No (*N* = 78,173)	Yes (*N* = 7141)	*p* value
Age(years), (median (IQR))	8.00 (4.00, 13.00)	11.00 (6.00, 15.00)	< 0.001	8.00 (4.00, 13.00)	13.00 (10.00, 15.00)	< 0.001	9.00 (4.00, 14.00)	12.00 (8.00, 15.00)	< 0.001	9.00 (4.00, 14.00)	8.00 (5.00, 13.00)	0.295
Maternal age (median (IQR))	30.00 (26.00, 34.00)	30.00 (25.00, 34.00)	< 0.001	31.00 (27.00, 34.00)	29.00 (24.00, 34.00)	< 0.001	30.00 (26.00, 34.00)	30.00 (25.00, 34.00)	< 0.001	30.00 (26.00, 34.00)	30.00 (26.00, 35.00)	0.388
Birth Weight (Ounces),(median (IQR))	118.00 (105.00, 130.00)	118.00 (103.00, 130.00)	0.13	118.00 (106.00, 130.00)	118.00 (103.00, 129.00)	< 0.001	118.00 (105.00, 130.00)	112.00 (88.00, 126.00)	< 0.001	118.00 (106.00, 130.00)	117.00 (102.00, 130.00)	< 0.001
Race, *n* (%)			0.001			< 0.001			0.001			< 0.001
Hispanic	64,091 (77.47)	1976 (76.56)		59,444 (77.08)	6623 (80.79)		65,427 (77.49)	640 (72.98)		60,506 (77.40)	5561 (77.87)	
White, non-Hispanic	5582 (6.75)	193 (7.48)		5164 (6.70)	611 (7.45)		5689 (6.74)	86 (9.81)		5237 (6.70)	538 (7.53)	
Black, non-Hispanic	768 (0.93)	18 (0.70)		725 (0.94)	61 (0.74)		774 (0.92)	12 (1.37)		702 (0.90)	84 (1.18)	
Asian, non-Hispanic	4557 (5.51)	108 (4.18)		4528 (5.87)	137 (1.67)		4623 (5.48)	42 (4.79)		4407 (5.64)	258 (3.61)	
American Indian or Alaska Native Non-Hispanic	560 (0.68)	18 (0.70)		541 (0.70)	37 (0.45)		570 (0.68)	8 (0.91)		534 (0.68)	44 (0.62)	
Multi-Race Non-Hispanic	7175 (8.67)	268 (10.38)		6714 (8.71)	729 (8.89)		7354 (8.71)	89 (10.15)		6787 (8.68)	656 (9.19)	
Gender, *n* (%)			< 0.001			< 0.001			< 0.001			< 0.001
Male	40,388 (48.82)	558 (21.62)		38,305 (49.67)	2641 (32.22)		40,629 (48.12)	317 (36.15)		38,609 (49.39)	2337 (32.73)	
Female	42,345 (51.18)	2023 (78.38)		38,811 (50.33)	5557 (67.78)		43,808 (51.88)	560 (63.85)		39,564 (50.61)	4804 (67.27)	
Concussion or Brain Injury, *n* (%)			< 0.001			< 0.001			< 0.001			< 0.001
No	78,321 (94.67)	2396 (92.83)		73,398 (95.18)	7319 (89.28)		79,949 (94.68)	768 (87.57)		74,132 (94.83)	6585 (92.21)	
Yes	4412 (5.33)	185 (7.17)		3718 (4.82)	879 (10.72)		4488 (5.32)	109 (12.43)		4041 (5.17)	556 (7.79)	
Preterm birth, *n* (%)			< 0.001			< 0.001			< 0.001			< 0.001
No	73,931 (89.36)	2147 (83.18)		69,148 (89.67)	6930 (84.53)		75,414 (89.31)	664 (75.71)		70,185 (89.78)	5893 (82.52)	
Yes	8802 (10.64)	434 (16.82)		7968 (10.33)	1268 (15.47)		9023 (10.69)	213 (24.29)		7988 (10.22)	1248 (17.48)	
Cerebral Palsy, *n* (%)			< 0.001			< 0.001			< 0.001			< 0.001
No	82,528 (99.75)	2546(98.64)		76,916 (99.74)	8158 (99.51)		84,276 (99.81)	798 (90.99)		78,076 (99.88)	6998(98.00)	
Yes	205 (0.25)	35 (1.36)		200 (0.26)	40 (0.49)		161 (0.19)	79 (9.01)		97 (0.12)	143 (2.00)	
Down Syndrome, *n* (%)			< 0.001			0.064			< 0.001			< 0.001
No	82,579 (99.81)	2556 (99.03)		76,962 (99.80)	8173 (99.70)		84,382 (99.93)	753 (85.86)		78,132 (99.95)	7003 (98.07)	
Yes	154 (0.19)	25 (0.97)		154 (0.20)	25 (0.30)		55 (0.07)	124 (14.14)		41 (0.05)	138 (1.93)	
Other genetic or inherited condition, *n* (%)			< 0.001			< 0.001			< 0.001			< 0.001
No	79,733 (96.37)	1920 (74.39)		74,614 (96.76)	7039 (85.86)		81,100 (96.05)	553 (63.06)		75,594 (96.70)	6059 (84.85)	
Yes	3000 (3.63)	661 (25.61)		2502 (3.24)	1159 (14.14)		3337 (3.95)	324 (36.94)		2579 (3.30)	1082 (15.15)	
Autism spectrum disorder (ASD), *n* (%)			NA			< 0.001			< 0.001			< 0.001
No	NA	NA		75,689 (98.15)	7044 (85.92)		82,251 (97.41)	482 (54.96)		77,018 (98.52)	5715 (80.03)	
Yes	NA	NA		1427 (1.85)	1154 (14.08)		2186 (2.59)	395 (45.04)		1155 (1.48)	1426 (19.97)	
Attention Deficit/Hyperactivity Disorder, *n* (%)			< 0.001			NA			< 0.001			< 0.001
No	75,689 (91.49)	1427 (55.29)		NA	NA		76,591 (90.71)	525 (59.86)		71,655 (91.66)	5461 (76.47)	
Yes	7044 (8.51)	1154 (44.71)		NA	NA		7846 (9.29)	352 (40.14)		6518 (8.34)	1680 (23.53)	
Intellectual Disability			< 0.001			< 0.001			NA			< 0.001
No	82,251 (99.42)	2186 (84.70)		76,591 (99.32)	7846 (95.71)		NA	NA		77,988 (99.76)	6449 (90.31)	
Yes	482 (0.58)	395 (15.30)		525 (0.68)	352 (4.29)		NA	NA		185 (0.24)	692 (9.69)	
Speech or language disorder, *n* (%)			< 0.001			< 0.001			< 0.001			NA
No	77,018 (93.09)	1155 (44.75)		71,655 (92.92)	6518 (79.51)		77,988 (92.36)	185 (21.09)		NA	NA	
Yes	5715 (6.91)	1426 (55.25)		5461 (7.08)	1680 (20.49)		6449 (7.64)	692 (78.91)		NA	NA	
Congenital Heart Condition, *n* (%)			< 0.001			< 0.001			< 0.001			< 0.001
No	80,889 (97.77)	2448 (94.85)		75,454 (97.84)	7883 (96.16)		82,599 (97.82)	738 (84.15)		76,617 (98.01)	6720 (94.10)	
Yes	1844 (2.23)	133 (5.15)		1662 (2.16)	315 (3.84)		1838 (2.18)	139 (15.85)		1556 (1.99)	421 (5.90)	
Heart Condition Currently, *n* (%)			< 0.001			< 0.001			< 0.001			< 0.001
Does not have condition	80,659 (97.49)	2435 (94.34)		75,251 (97.58)	7843 (95.67)		82,363 (97.54)	731 (83.35)		76,404 (97.74)	6690 (93.68)	
Ever, but not currently	1123 (1.36)	90 (3.49)		1012 (1.31)	201 (2.45)		1127 (1.33)	86 (9.81)		964 (1.23)	249 (3.49)	
Currently has condition	951 (1.15)	56 (2.17)		853 (1.11)	154 (1.88)		947 (1.12)	60 (6.84)		805 (1.03)	202 (2.83)	
Severity of Current Heart Condition, *n* (%)			< 0.001			< 0.001			< 0.001			< 0.001
Does not currently have condition	81,610 (98.64)	2491 (96.51)		76,104 (98.69)	7997 (97.55)		83,310 (98.67)	791 (90.19)		77,209 (98.77)	6892 (96.51)	
Mild	904 (1.09)	65 (2.52)		819 (1.06)	150 (1.83)		914 (1.08)	55 (6.27)		791 (1.01)	178 (2.49)	
Moderate	166 (0.20)	20 (0.77)		142 (0.18)	44 (0.54)		160 (0.19)	26 (2.96)		135 (0.17)	51 (0.71)	
Severe	53 (0.06)	5 (0.19)		51 (0.07)	7 (0.09)		53 (0.06)	5 (0.57)		38 (0.05)	20 (0.28)	

Abbreviations: IQR, interquartile range; NA, not applicable.

**Table 2:** The association between congenital heart condition and the risk of neurodevelopmental disorders in multivariable regression analyses and propensity score matching

Variable	Autism spectrum disorder			Attention Deficit/Hyperactivity Disorder			Intellectual disability			Speech or language disorder		
	**Multivariable regression analyses**											
	**OR (95% CI)**	***P value***		**OR (95% CI)**	***P value***		**OR (95% CI)**	***P value***		**OR (95% CI)**	***P value***	
Congenital Heart Condition												
No	Reference			Reference			Reference			Reference		
Yes	0.95(0.26–1.64)	0.88		1.01(0.62–1.4)	0.96		1.62(0.56–2.68)	0.37		1.61(1.14–2.08)	0.04	
Heart Condition Currently												
Does not have condition	Reference			Reference			Reference			Reference		
Ever, but not currently	0.81(-0.5-2.12)	0.76		0.64(-0.36-1.64)	0.38		0.98(-0.69-2.65)	0.99		2.45(1.65–3.25)	0.03	
Currently has condition	0.94(0.23–1.65)	0.87		1.22(0.83–1.61)	0.32		0.75(-0.35-1.85)	0.6		1.18(0.71–1.65)	0.49	
Severity of Current Heart Condition												
Does not currently have condition	Reference			Reference			Reference			Reference		
Mild	1.38(0.2–2.56)	0.59		1.92(0.98–2.86)	0.17		1.02(-0.33- 2.37)	0.98		0.4(-0.27- 1.07)	0.01	
Moderate	1.41(0.14–2.68)	0.6		2.4(1.4–3.4)	0.08		1.71(0.24–3.18)	0.47		0.4(-0.36- 1.16)	0.02	
Severe	NA	NA		NA	NA		NA	NA		NA	NA	
	**Propensity score matching**											
	**No (** *N* ** = 2510)**	**Yes (** *N* ** = 2510)**	***P value***	**No (** *N* ** = 8063)**	**Yes (** *N* ** = 8063)**	***P value***	**No (** *N* ** = 780)**	**Yes (** *N* ** = 780)**	***P value***	**No (** *N* ** = 6492)**	**Yes (** *N* ** = 6492)**	***P value***
Congenital Heart Condition, *n* (%)												
No	2374 (94.6)	2387 (95.1)	0.444	7826 (97.1)	7757 (96.2)	0.003	717 (91.9)	686 (87.9)	0.012	6296 (97.0)	6177 (95.1)	< 0.001
Yes	136 (5.4)	123 (4.9)		237 (2.9)	306 (3.8)		63 (8.1)	94 (12.1)		196 (3.0)	315 ( 4.9)	
Heart Condition Currently, *n* (%)												
Does not have condition	2365 (94.2)	2375 (94.6)	0.698	7803 (96.8)	7718 (95.7)	0.002	711 (91.2)	680 (87.2)	0.033	6273 (96.6)	6153 (94.8)	< 0.001
Ever, but not currently	82 (3.3)	81 (3.2)		152 (1.9)	196 (2.4)		42 ( 5.4)	66 (8.5)		126 (1.9)	188 (2.9)	
Currently has condition	63 (2.5)	54 (2.2)		108 (1.3)	149 (1.8)		27 (3.5)	34 (4.4)		93 (1.4)	151 (2.3)	
Severity of Current Heart Condition, *n* (%)												
Does not currently have condition	2428 (96.7)	2429 (96.8)	< 0.001	7911 (98.1)	7867 (97.6)	< 0.001	738 (94.6)	714 (91.5)	0.033	6366 (98.1)	6304 (97.1)	0.001
Mild	59 (2.4)	58 (2.3)		113 (1.4)	145 (1.8)		30 (3.8)	42 (5.4)		99 (1.5)	136 (2.1)	
Moderate	18 (0.7)	19 (0.8)		26 (0.3)	44 (0.5)		7 (0.9)	20 (2.6)		23 (0.4)	36 (0.6)	
Severe	5 (0.2)	4 (0.2)		13 (0.2)	7 ( 0.1)		5 (0.6)	4 (0.5)		4 (0.1)	16 (0.2)	

Abbreviations: OR, odds ratio; CI, confidence interval; NA, not applicable.

To address possible bias caused by confounding variables, we constructed a 1:1 PSM model. After including these covariates in the model, we successfully matched 2,510 pairs for the ASD group, 8,063 pairs for the ADHD group, 780 pairs for the intellectual disability group, and 6,492 pairs for the speech or language disorder group. As shown in Supplementary Table 4, most baseline characteristics did not have statistically significant differences between the groups (*p* > 0.05), indicating that the PS-matched samples achieved balance. For ASD, the severity of current heart condition (*p* < 0.001) had a significant association with it in PS-matched samples (Table 2 and Supplementary Table 4). For ADHD, the variables of congenital heart disease (*p* = 0.003), heart condition currently (*p* = 0.002), and severity of current heart condition (*p* < 0.001) were all associated with it. Similarly, intellectual disability was significantly related to congenital heart disease (*p* = 0.012), heart condition currently (*p* = 0.033), and severity of current heart condition(*p* = 0.033). For speech or language disorders, congenital heart disease (*p* < 0.001), heart condition currently (*p* < 0.001), and severity of current heart condition (*p* = 0.001) were also significantly associated with it, consistent with the result of multivariable regression analysis.

### MR analysis

**Fig. 2:**
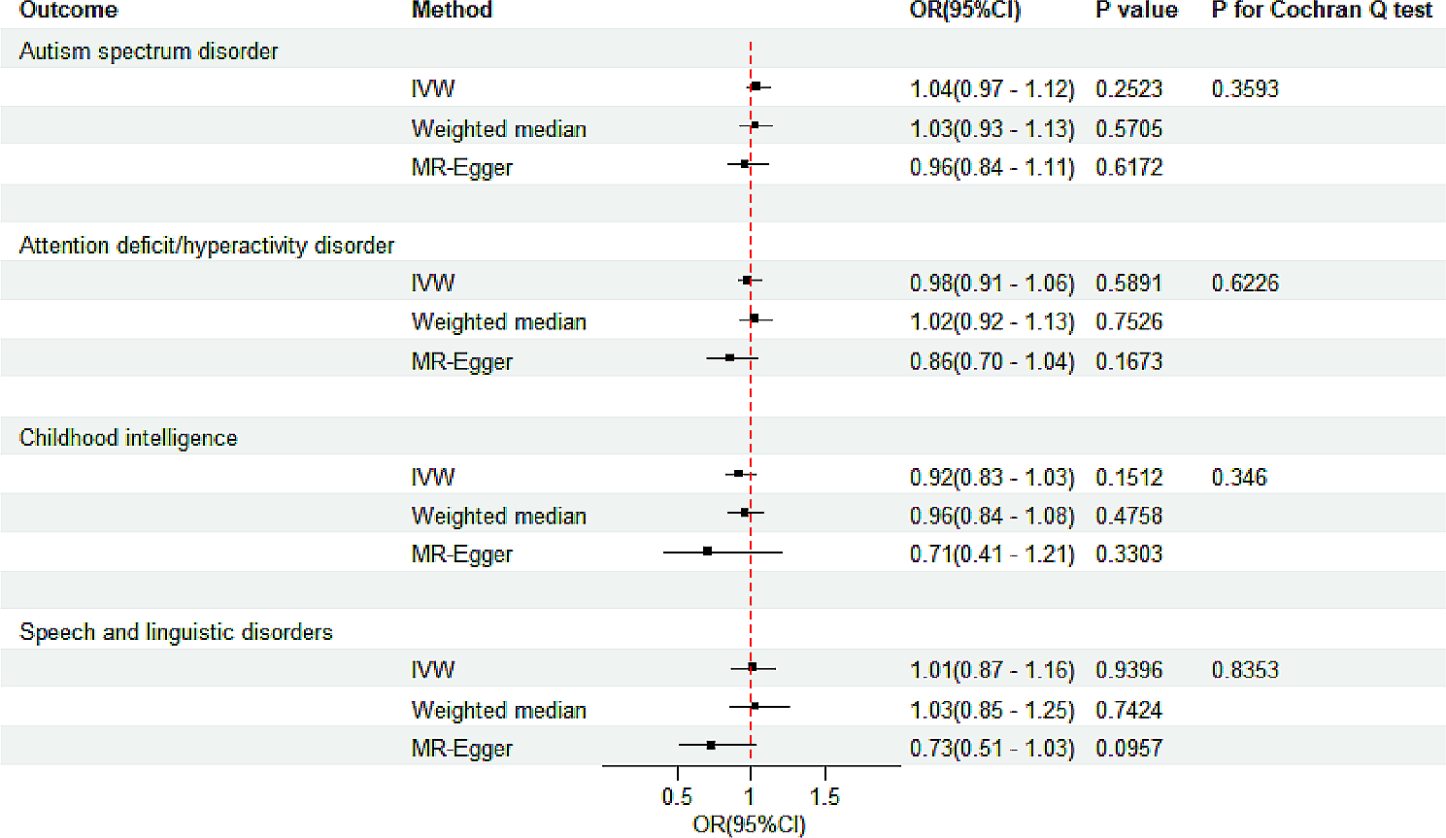
Association of genetically proxied congenital cardiac malformations with four neurodevelopmental disorders. Odds ratios are per standard deviation increment in the exposure. IVW, inverse-variance-weighted; MR, Mendelian randomization

IVW-MR analysis did not find a causal relationship between genetically proxied congenital cardiac malformations and the risk of NDDs. This result was consistent with the findings from the weighted median and MR-Egger methods (Fig. 2 and Supplementary Table 5). The F-statistics of all SNPs used as instrument variants were > 20, indicating no significant weak instrument bias (Supplementary Table 1). Additionally, sensitivity and heterogeneity analyses demonstrated that there was no significant evidence of pleiotropy or heterogeneity (Supplementary Table 6), indicating the stability and reliability of the MR results. The leave-one‐out analysis and scatter plots also did not identify any outlying SNPs (Supplementary Figs. 1–4).

## Discussion

In this large, nationally representative study, our results provided positive evidence of an association between CHD and NDDs through regression analysis and PSM. In addition, our results also found that heart condition currently and the severity of the current heart condition are significantly associated with these neurodevelopmental disorders.

Our results are consistent with some of the previous studies [4–13]. Previous small sample studies have already found that children with left ventricular hypoplasia syndromes have a significantly higher risk of being diagnosed with autism than children without CHD [4–6]. Specifically, 4 out of 58 children with left ventricular hypoplasia syndrome were diagnosed with ASD, while none of the control group were diagnosed. However, Wier et al. [25] a retrospective case–control study (ASD *n* = 417; control *n* = 2,067) yielded a contradictory report. They did not find a significant association between ASD and cardiac defects (OR = 1.5, 95% CI = 0.7–2.8). It is possible that this study were not well remove the influence of confounding factors on the results, especially they include all congenital malformations.

To address this potential reporting bias, Tsao et al. [8] conducted a retrospective case-control study with 3,552 CHD cases and 14,208 Non-CHD controls. Multivariate Cox regression analysis showed that children with CHD had an increased risk of developing ADHD (hazard ratio [HR] 2.52, 95% CI 1.96–3.25) and ASD (HR 1.97, 95% CI 1.11–3.52). Recently, using a large Military Health System (MHS) administrative database with 8,760 autism spectrum disorders cases and 26,280 controls, Sigman et al. [9] also demonstrated an increased odds of ASD in patients with CHD (OR 1.32; 95% CI 1.10–1.59). However, these previous studies only focused on the association between developmental delays and CHD but did not clarify the causal relationship between them.

To further obtain causal associations, we conducted a two-sample MR study. However, MR analysis did not find causality between genetically proxied congenital cardiac malformations and the risk of NDDs. This result of MR may seem counterintuitive at first sight when compared to our prior studies. Therefore, some interpretations of this contradictory finding should be listed before drawing our final conclusions.

First, in MR studies, we did not include the impact of external intervention measures (such as surgery). This is necessary for the survival of children, but it may also complicate neurodevelopment. These interventions generate various factors, including preoperative and postoperative brain injury and intraoperative hemodynamic factors, among others [11, 12, 26]. Indeed, previous studies have largely identified the significant impact of surgery on neurodevelopmental outcomes in children. However, because the genetic effect is lifelong, the MR study cannot estimate the impact of intervention measures at a specific time in life. This may result in the effect sizes of exposure inferred by genetic analysis being different from those in reality. In this MR study, we only explored the impact of congenital heart malformation itself on children, which may imply that the impact of external interventions such as surgery on neurological development may not be significantly reflected.

Second, pleiotropy in genetic loci may contribute to the risk of both disorders, but it needs to be avoided in MR analysis. During early morphogenesis, the brain shares the same genetic pathway with the heart, and the genetic factors that cause heart malformations may also disrupt the normal expression of gene fragments for brain development, thereby triggering congenital brain injury and NDDs [27]. In 2015, a meta-analysis showed that the incidence of CHARGE syndrome with ASD was 30%, and the incidence of 22q11.2 deletion syndrome with ASD was 11% [28]. Similarly, a review by Vorstman et al. [29] summarizes that at least 10 chromosomal abnormalities can lead to heart defects in patients with ASD. Genetic factors are an important cause of NDDs in children with CHD. However, in the MR study, all genetic variations with pleiotropic effects on the outcome cannot be used as instrumental variables for proxy exposure [30]. Instrument variables can only affect the outcome through exposure instead of other pathways [31]. This exclusivity assumption is essential for the MR design to ensure the validity of the causal assessment. Therefore, our results of MR did not preclude an association between both disorders, as cardiac malformations may increase the risk of NDDs *via* genetic factors.

Thirdly, it should be emphasized that genetic changes are also important in CHD and NDDs. Recent studies have shown that genes related to autism with damaging *de novo* mutations have been identified in patients with CHD, which highlighting the overlap between the development of the heart and neurodevelopment [13, 32]. However, the penetrance of these genetic mutations (include point mutations, copy number variations, and aneuploidy) varies. And their contribution to NDDs in patients with CHD remains unclear. Therefore, it is difficult to accurately evaluate this factor or conduct layered analysis in MR study, which may partly explain the contradiction results between our research. In summary, although the MR study did not fully elucidate causality between CHD and NDDs, it does not mean denying the impact of CHD on children’s neurodevelopment. In the future, controlled trials and longitudinal follow-up cohorts are needed to infer causality between them.

As discussed previously, our study shows that children with CHD have an increased risk of developing NDDs. The heart conditions currently and severity of current heart conditions were also significantly associated with these NDDs. This means that compared with normal children, the quality of life and social participation ability of CHD population may be lower than expected, and the burden of mental health is higher, especially in children with severe cardiac malformations. Therefore, pediatricians should not only pay attention to the circulation function of children, but also pay attention to the impact of the complexity of heart disease on their neurodevelopment. For children with severe cardiac malformations, comprehensive neurodevelopmental evaluation or treatment should be provided as soon as possible. In addition, CHD children should also undergo routine neurodevelopmental screening and regular follow-up, so that each CHD child with NDDs can benefit from timely and effective individualized rehabilitation treatment or special education according to their condition, and ultimately improve the neurodevelopmental prognosis of CHD children and the quality of life.

The advantages of our study include the use of a large, nationally representative dataset and the sample was selected from nationwide households instead of the medical system. As a result, our results are less biased and can be generalized to a larger population of children and adolescents. Additionally, we conducted an MR study using summary-level genome-wide association study data. MR analysis can overcome some limitations of observational studies, such as measurement or confounding errors, and facilitate evaluating the long-term effects of cardiac malformation.

There are some limitations of this study. First of all, we cannot rule out that we may have overlooked some associations due to inadequate statistical power. Although our research samples are large, the number of children with complex heart malformations is still not enough to perform some statistical analysis. In addition, the lack of causality in MR research does not exclude the biological effect of CHD, but rather indicates a lack of evidence that changes in the expression of genes are related to NDDs. Additionally, the measurement of some covariates was based on parental reports in our observational study. However, parental reports may be prejudiced or may not accurately reflect the child’s health status. Finally, the genetic summary data we used for the MR study were derived from populations with European ancestry, which may limit the universality of our results in other races.

## Conclusions

Our study shows that children with CHD have an increased risk of developing NDDs. Heart conditions currently and severity of current heart conditions were also significantly associated with these neurodevelopmental disorders. In the future, we need to try more methods to clarify the causal relationship between CHD and NDDs.

### Electronic supplementary material

Below is the link to the electronic supplementary material.

Supplementary Material 1

## Data Availability

The National Survey of Children’s Health (2020–2021) data are available from: http://childhealthdata.org/learn/NSCH. Genome-wide association studies summary-level statistics were accessed through the IEU OpenGWAS project (https://gwas.mrcieu.ac.uk/) and the FinnGen study (https://finngen.gitbook.io/documentation/data-download).

## References

[1] Gilboa SM, Salemi JL, Nembhard WN, Fixler DE, Correa A (2010). Mortality resulting from congenital heart disease among children and adults in the United States, 1999 to 2006. Circulation.

[2] Oster ME, Lee KA, Honein MA, Riehle-Colarusso T, Shin M, Correa A (2013). Temporal trends in survival among infants with critical congenital heart defects. Pediatrics.

[3] Marino BS, Lipkin PH, Newburger JW (2012). Neurodevelopmental outcomes in children with congenital heart disease: evaluation and management: a scientific statement from the American Heart Association. Circulation.

[4] Brosig CL, Bear L, Allen S (2018). Neurodevelopmental outcomes at 2 and 4 years in children with congenital heart disease. Congenit Heart Dis.

[5] von Rhein M, Dimitropoulos A, Valsangiacomo Buechel ER, Landolt MA, Latal B (2012). Risk factors for neurodevelopmental impairments in school-age children after cardiac surgery with full-flow cardiopulmonary bypass. J Thorac Cardiovasc Surg.

[6] Newburger JW, Sleeper LA, Bellinger DC (2012). Early developmental outcome in children with hypoplastic left heart syndrome and related anomalies: the single ventricle reconstruction trial. Circulation.

[7] Ricci MF, Andersen JC, Joffe AR (2015). Chronic Neuromotor Disability after Complex Cardiac surgery in early life. Pediatrics.

[8] Tsao PC, Lee YS, Jeng MJ (2017). Additive effect of congenital heart disease and early developmental disorders on attention-deficit/hyperactivity disorder and autism spectrum disorder: a nationwide population-based longitudinal study. Eur Child Adolesc Psychiatry.

[9] Sigmon ER, Kelleman M, Susi A, Nylund CM, Oster ME (2019). Congenital heart disease and autism: a case-control study. Pediatrics.

[10] Cassidy AR, Newburger JW, Bellinger DC (2017). Learning and memory in adolescents with critical biventricular congenital heart disease. J Int Neuropsychol Soc.

[11] Bergemann A, Hansen JH, Rotermann I (2015). Neuropsychological performance of school-aged children after staged surgical palliation of hypoplastic left heart syndrome. Eur J Cardiothorac Surg.

[12] Sananes R, Manlhiot C, Kelly E (2012). Neurodevelopmental outcomes after open heart operations before 3 months of age. Ann Thorac Surg.

[13] Homsy J, Zaidi S, Shen Y (2015). De novo mutations in congenital heart disease with neurodevelopmental and other congenital anomalies. Science.

[14] Davies NM, Holmes MV, Davey Smith G (2018). Reading mendelian randomisation studies: a guide, glossary, and checklist for clinicians. BMJ.

[15] Smith GD, Ebrahim S. ‘Mendelian randomization’: can genetic epidemiology contribute to understanding environmental determinants of disease? Int J Epidemiol. 2003;32(1):1–22. 10.1093/ije/dyg070. 10.1093/ije/dyg070 12689998

[16] Health R. & Services Administration, Maternal & Child Health Bureau. (n.d.). https://mchb.hrsa.gov/.

[17] Data Resource Center for Child. & Adolescent Health (n.d.). The national survey of children’s health.http://childhealthdata.org/learn/NSCH.

[18] Grove J, Ripke S, Als TD (2019). Identification of common genetic risk variants for autism spectrum disorder. Nat Genet.

[19] Demontis D, Walters RK, Martin J (2019). Discovery of the first genome-wide significant risk loci for attention deficit/hyperactivity disorder. Nat Genet.

[20] Benyamin B, Pourcain B, Davis OS (2014). Childhood intelligence is heritable, highly polygenic and associated with FNBP1L. Mol Psychiatry.

[21] Burgess S, Thompson SG, CRP CHD Genetics Collaboration (2011). Avoiding bias from weak instruments in mendelian randomization studies. Int J Epidemiol.

[22] Burgess S, Thompson SG. Interpreting findings from Mendelian randomization using the MR-Egger method [published correction appears in Eur J Epidemiol. 2017;:]. Eur J Epidemiol. 2017;32(5):377–389. 10.1007/s10654-017-0255-x. 10.1007/s10654-017-0255-x PMC5506233 28527048

[23] Verbanck M, Chen CY, Neale B, Do R. Detection of widespread horizontal pleiotropy in causal relationships inferred from Mendelian randomization between complex traits and diseases [published correction appears in Nat Genet. 2018;50(8):1196]. Nat Genet. 2018;50(5):693–698. 10.1038/s41588-018-0099-7. 10.1038/s41588-018-0099-7 PMC6083837 29686387

[24] Higgins JP, Thompson SG, Deeks JJ, Altman DG (2003). Measuring inconsistency in meta-analyses. BMJ.

[25] Wier ML, Yoshida CK, Odouli R, Grether JK, Croen LA (2006). Congenital anomalies associated with autism spectrum disorders. Dev Med Child Neurol.

[26] Dent CL, Spaeth JP, Jones BV (2005). Brain magnetic resonance imaging abnormalities after the Norwood procedure using regional cerebral perfusion. J Thorac Cardiovasc Surg.

[27] McQuillen PS, Goff DA, Licht DJ (2010). Effects of congenital heart disease on brain development. Prog Pediatr Cardiol.

[28] Richards C, Jones C, Groves L, Moss J, Oliver C (2015). Prevalence of autism spectrum disorder phenomenology in genetic disorders: a systematic review and meta-analysis. Lancet Psychiatry.

[29] Vorstman JAS, Parr JR, Moreno-De-Luca D, Anney RJL, Nurnberger JI, Hallmayer JF (2017). Autism genetics: opportunities and challenges for clinical translation. Nat Rev Genet.

[30] Sanderson E (2021). Multivariable mendelian randomization and mediation. Cold Spring Harb Perspect Med.

[31] Paternoster L, Tilling K, Davey Smith G. Genetic epidemiology and Mendelian randomization for informing disease therapeutics: Conceptual and methodological challenges. PLoS Genet. 2017;13(10):e1006944. Published 2017 Oct 5. 10.1371/journal.pgen.1006944. 10.1371/journal.pgen.1006944 PMC5628782 28981501

[32] Jin SC, Homsy J, Zaidi S (2017). Contribution of rare inherited and de novo variants in 2,871 congenital heart disease probands. Nat Genet.

