# Supplementary material for: Association of congenital heart disease and neurodevelopmental disorders: an observational and Mendelian randomization study
Source: Ital J Pediatr. 2024 Apr 8;50:63. doi: 10.1186/s13052-024-01610-3 (PMC11003105; doi:10.1186/s13052-024-01610-3)

Supplementary Materials

Supplementary Table 1. Information of single nucleotide polymorphisms used as instrumental variables.

Supplementary Table 2. Information of GWAS summary data used in the present study.

Supplementary Table 3. Multivariable logistic regression analyses of potential risk factors for neurodevelopmental disorders.

Supplementary Table 4. Baseline characteristics of children according to the presence of neurodevelopmental disorders after propensity score matching.

Supplementary Table 5. Association between genetically predicted congenital heart disease and neurodevelopmental disorders using inverse-variance weighted, Weighted median and MR-Egger methods.

Supplementary Table 6. Heterogeneity and sensitivity analyses in Mendelian randomization study using Cochran Q test, MR-Egger and MR-PRESSO methods.

Supplementary Figure 1. The scatter plot and results of leave-one-out analysis for autism spectrum disorder.

Supplementary Figure 2. The scatter plot and results of leave-one-out analysis for attention-deficit/hyperactivity disorder.

Supplementary Figure 3. The scatter plot and results of leave-one-out analysis for intellectual disability.

Supplementary Figure 4. The scatter plot and results of leave-one-out analysis for speech or language disorder.

Supplementary Table 1. Information of single nucleotide polymorphisms used as instrumental variables.

SNP	Chr	Base pair	Effect allele	Other allele	Effect allele frequency	Beta	SE	p value	F-statistic
rs7543039	1	99584092	T	C	0.481595	0.120299	0.0242475	7.00E-07	24.6144
rs186619278	1	184131190	G	A	0.0362712	0.273096	0.0592599	4.06E-06	21.2378
rs72809964	2	73273924	A	G	0.021546	0.358721	0.0764614	2.71E-06	22.0105
rs34485446	2	84845558	A	G	0.211535	0.142587	0.0291128	9.70E-07	23.9879
rs729327	3	11132638	A	G	0.0705466	0.235755	0.0444073	1.10E-07	28.1847
rs1319007	4	4665619	C	G	0.24914	0.150933	0.0273075	3.25E-08	30.5495
rs192987035	6	7558933	T	A	0.0105896	0.516519	0.101434	3.54E-07	25.9302
rs12663370	6	33639596	G	T	0.150188	-0.161116	0.0351552	4.58E-06	21.0038
rs139210991	7	25032010	C	T	0.0136711	-0.60829	0.128893	2.37E-06	22.2722
rs3087368	7	44114893	T	C	0.0257437	0.325994	0.0703122	3.55E-06	21.4960
rs61506866	10	5080610	T	G	0.208532	0.141746	0.0292018	1.21E-06	23.5615
rs17686420	16	20142191	T	C	0.00215158	0.952859	0.20387	2.96E-06	21.8449
rs6504672	17	47019579	A	T	0.17497	0.195794	0.0305003	1.37E-10	41.2089
rs2958659	22	40004908	G	A	0.291974	-0.133992	0.0270429	7.24E-07	24.5500

Abbreviations: SNP, single nucleotide polymorphism; Chr, chromosome; SE, standard error.

Supplementary Table 2. Information of GWAS summary data used in the present study.

Traits	Dataset source	Sample size	Population ancestry	PMID	Authors	Data download
Congenital malformations of heart and great arteries	FinnGen	Number of cases:3,459 Number of controls:33,9040	European	NA	NA	https://storage.googleapis.com/finngen-public-data-r8/summary_stats/finngen_R8_CONGEN_HEART_ARTER.gz
Autism Spectrum Disorder	iPSYCH(Integrative Psychiatric Research)-PGC	Number of cases:18,382 Number of controls:27,969	European	30804558	Grove, Jakob et al	https://gwas.mrcieu.ac.uk/datasets/ieu-a-1185/
Attention deficit/hyperactivity disorder	Psychiatric Genomics Consortium (PGC)	Number of cases:20,183 Number of controls:35,191	European	30478444	Demontis, Ditte et al	https://gwas.mrcieu.ac.uk/datasets/ieu-a-1183/
Childhood intelligence	Social Science Genetic Association Consortium(SSGAC)	Number of Sample sizes: 12,441	European	23358156	Benyamin, B et al	https://gwas.mrcieu.ac.uk/datasets/ieu-a-16/
Speech and linguistic disorders	FinnGen	Number of cases:2,288 Number of controls:25,4976	European	NA	NA	https://storage.googleapis.com/finngen-public-data-r8/summary_stats/finngen_R8_KRA_PSY_SPEELING_EXMORE.gz

Abbreviations: GWAS, genome-wide association study; NA, not applicable.

Supplementary Table 3. Multivariable logistic regression analyses of potential risk factors for neurodevelopmental disorders.

Variable	Autism spectrum disorder		Attention Deficit/Hyperactivity Disorder		Intellectual Disability		Speech or language disorder	
	Multivariate Adjusted OR (95% CI)	P value	Multivariate Adjusted OR (95% CI)	P value	Multivariate Adjusted OR (95% CI)	P value	Multivariate Adjusted OR (95% CI)	P value
Age(years), (median [IQR])	1.05(1.05-1.05)	<0.001	1.18(1.18-1.18)	<0.001	1.11(1.09-1.13)	<0.001	1(1-1)	0.02
Maternal age(median [IQR])	1(1-1)	0.63	0.96(0.96-0.96)	<0.001	0.97(0.95-0.99)	<0.001	NA	NA
Birth Weight(Ounces),(median [IQR])	1(1-1)	0.05	1(1-1)	<0.001	0.99(0.99-0.99)	<0.001	NA	NA
Race, n (%)								
Hispanic	Reference		Reference		Reference		Reference	
White, non-Hispanic	NA	NA	0.95(0.85-1.05)	0.26	NA	NA	NA	NA
Black, non-Hispanic	NA	NA	0.57(0.3-0.84)	<0.001	NA	NA	NA	NA
Asian, non-Hispanic	NA	NA	0.28(0.1-0.46)	<0.001	NA	NA	NA	NA
American Indian or Alaska Native Non-Hispanic	NA	NA	0.53(0.18-0.88)	<0.001	NA	NA	NA	NA
Multi-Race Non-Hispanic	NA	NA	1.02(0.94-1.1)	0.6	NA	NA	NA	NA
Gender, n (%)								
Male	Reference		Reference		Reference		Reference	
Female	2.67(2.57-2.77)	<0.001	1.94(1.88-2)	<0.001	0.95(0.77-1.13)	0.58	1.72(1.66-1.78)	<0.001
Concussion or Brain Injury, n (%)								
No	Reference		Reference		Reference		Reference	
Yes	0.64(0.46-0.82)	<0.001	1.27(1.19-1.35)	<0.001	1.3(1.05-1.55)	0.05	1.19(1.09-1.29)	<0.001
Preterm birth, n (%)								
No	Reference		Reference		Reference		Reference	
Yes	1.22(1.08-1.36)	0.01	1.31(1.23-1.39)	<0.001	1.07(0.83-1.31)	0.57	1.49(1.41-1.57)	<0.001
Cerebral Palsy, n (%)								
No	Reference		Reference		Reference		Reference	
Yes	0.68(0.25-1.11)	0.08	0.53(0.14-0.92)	<0.001	17.21(16.84-17.58)	<0.001	6.66(6.35-6.97)	<0.001
Down Syndrome, n (%)								
No	Reference		Reference		Reference		Reference	
Yes	0.29(-0.24-0.82)	<0.001	NA	NA	149.79(149.34-150.24)	<0.001	8.73(8.3-9.16)	<0.001
Other genetic or inherited condition, n (%)								
No	Reference		Reference		Reference		Reference	
Yes	3.27(3.15-3.39)	<0.001	3.03(2.95-3.11)	<0.001	3.73(3.55-3.91)	<0.001	2.57(2.47-2.67)	<0.001
Intellectual Disability								
No	Reference		Reference		Reference		Reference	
Yes	5.23(5.03-5.43)	<0.001	1.17(0.99-1.35)	0.08	NA	NA	10.96(10.76-11.16)	<0.001
Speech or language disorder, n								

(%)								
No	Reference		Reference		Reference		Reference	
Yes	9.44(9.34-9.54)	<0.001	2.19(2.11-2.27)	<0.001	15.26(15.06-15.46)	<0.001	NA	NA
Autism spectrum disorder(ASD), n (%)								
No	Reference		Reference		Reference		Reference	
Yes	NA	NA	3.91(3.81-4.01)	<0.001	6.18(6-6.36)	<0.001	8.35(8.25-8.45)	<0.001
Attention Deficit/Hyperactivity Disorder(ADHD), n (%)								
No	Reference		Reference		Reference		Reference	
Yes	4.05(3.95-4.15)	<0.001	NA	NA	1.81(1.63-1.99)	<0.001	1.75(1.67-1.83)	<0.001
Congenital Heart Condition, n (%)								
No	Reference		Reference		Reference		Reference	
Yes	0.95(0.26-1.64)	0.88	1.01(0.62-1.4)	0.96	1.62(0.56-2.68)	0.37	1.61(1.14-2.08)	0.04
Heart Condition Currently, n (%)								
Does not have condition	Reference		Reference		Reference		Reference	
Ever, but not currently	0.81(-0.5-2.12)	0.76	0.64(-0.36-1.64)	0.38	0.98(-0.69-2.65)	0.99	2.45(1.65-3.25)	0.03
Currently has condition	0.94(0.23-1.65)	0.87	1.22(0.83-1.61)	0.32	0.75(-0.35-1.85)	0.6	1.18(0.71-1.65)	0.49
Severity of Current Heart Condition, n (%)								
Does not currently have condition	Reference		Reference		Reference		Reference	
Mild	1.38(0.2-2.56)	0.59	1.92(0.98-2.86)	0.17	1.02(-0.33-2.37)	0.98	0.4(-0.27-1.07)	0.01
Moderate	1.41(0.14-2.68)	0.6	2.4(1.4-3.4)	0.08	1.71(0.24-3.18)	0.47	0.4(-0.36-1.16)	0.02
Severe	NA	NA	NA	NA	NA	NA	NA	NA

Abbreviations: IQR, interquartile range; OR, odds ratio; CI,confidence interval; NA, not applicable.

Supplementary Table 4. Baseline characteristics of children according to the presence of neurodevelopmental disorders after propensity score matching.

Characteristics	Autism spectrum disorder			Attention Deficit/Hyperactivity Disorder			Intellectual Disability			Speech or language disorder		
	No (N = 2510)	Yes (N = 2510)	p value	No (N = 8063)	Yes (N = 8063)	p value	No (N = 780)	Yes (N = 780)	p value	No (N = 6492)	Yes (N = 6492)	p value
Age(years), (median (IQR))	11.00 (7.00, 15.00)	11.00 (6.00, 15.00)	0.045	14.00 (10.00, 16.00)	13.00 (10.00, 15.00)	<0.001	11.50 (8.00, 15.00)	12.00 (8.00, 15.00)	0.957	9.00 (5.00, 13.00)	8.00 (5.00, 12.00)	0.005
Maternal age(median (IQR))	30.00 (25.00, 34.00)	30.00 (25.00, 34.00)	0.489	29.00 (25.00, 33.00)	29.00 (24.00, 34.00)	0.897	29.00 (24.00, 34.00)	29.00 (24.00, 34.00)	0.508	30.00 (26.00, 34.00)	30.00 (26.00, 34.00)	0.836
Birth Weight(Ounces),(median (IQR))	119.00 (103.00, 131.00)	118.00 (103.25, 130.00)	0.513	118.00 (104.00, 129.00)	118.00 (103.00, 129.00)	0.313	110.00 (89.00, 123.00)	112.00 (91.00, 127.00)	0.04	118.00 (104.00, 130.00)	118.00 (103.00, 130.00)	0.497
Race, n (%)			0.685			0.324			0.766			<0.001
Hispanic	1964 (78.2)	1929 (76.9)		6597 (81.8)	6505 (80.7)		581 (74.5)	573 (73.5)		5270 (81.2)	5087 (78.4)	
White, non-Hispanic	194 (7.7)	186 (7.4)		604 (7.5)	605 (7.5)		82 (10.5)	74 (9.5)		440 (6.8)	469 (7.2)	
Black, non-Hispanic	15 (0.6)	18 (0.7)		61 (0.8)	61 (0.8)		10 (1.3)	10 (1.3)		65 (1.0)	74 (1.1)	
Asian, non-Hispanic	93 (3.7)	106 (4.2)		116 (1.4)	137 (1.7)		25 (3.2)	35 (4.5)		166 (2.6)	234 (3.6)	
American Indian or Alaska Native Non-Hispanic	13 (0.5)	16 (0.6)		35 (0.4)	37 (0.5)		5 (0.6)	7 (0.9)		24 (0.4)	39 (0.6)	
Multi-Race Non-Hispanic	231 (9.2)	255 (10.2)		650 (8.1)	718 (8.9)		77 (9.9)	81 (10.4)		527 (8.1)	589 (9.1)	
Gender, n (%)			0.39			1			0.79			<0.001
Male	525 (20.9)	551 (22)		2628 (32.6)	2627 (32.6)		267 (34.2)	273 (35.0)		1977 (30.5)	2171 (33.4)	
Female	1985 (79.1)	1959 (78.0)		5435 (67.4)	5436 (67.4)		513 (65.8)	507 (65.0)		4515 (69.5)	4321 (66.6)	
Concussion or Brain Injury, n (%)			1			0.424			0.76			0.483
No	2332 (92.9)	2331 (92.9)		7238 (89.8)	7206 (89.4)		680 (87.2)	685 (87.8)		5997 (92.4)	6019 (92.7)	
Yes	178 (7.1)	179 (7.1)		825 (10.2)	857 (10.6)		100 (12.8)	95 (12.2)		495 (7.6)	473 (7.3)	
Preterm birth, n (%)			0.348			0.023			0.081			0.454
No	2069 (82.4)	2095 (83.5)		6935 (86.0)	6832 (84.7)		567 (72.7)	598 (76.7)		5376 (82.8)	5409 (83.3)	
Yes	441 (17.6)	415 (16.5)		1128 (14.0)	1231 (15.3)		213 (27.3)	182 (23.3)		1116 (17.2)	1083 (16.7)	
Cerebral Palsy, n (%)			0.904			0.826			0.701			0.733
No	2474 (98.6)	2476 (98.6)		8020 (99.5)	8023 (99.5)		724 (92.8)	719 (92.2)		6425 (99.0)	6420 (98.9)	
Yes	36 (1.4)	34 (1.4)		43 (0.5)	40 (0.5)		56 (7.2)	61 (7.8)		67 (1.0)	72 (1.1)	
Down Syndrome, n (%)			0.494			0.888			0.144			0.487
No	2480 (98.8)	2486 (99)		8037 (99.7)	8039 (99.7)		745 (95.5)	731 (93.7)		6458 (99.5)	6451 (99.4)	
Yes	30 (1.2)	24 (1.0)		26 (0.3)	24 (0.3)		35 (4.5)	49 (6.3)		34 (0.5)	41 (0.6)	
Other genetic or inherited condition, n (%)			0.066			<0.001			0.462			0.651
No	1953 (77.8)	1897 (75.6)		7139 (88.5)	6991 (86.7)		503 (64.5)	488 (62.6)		5676 (87.4)	5694 (87.7)	
Yes	557 (22.2)	613 (24.4)		924 (11.5)	1072 (13.3)		277 (35.5)	292 (37.4)		816 (12.6)	798 (12.3)	
Autism spectrum disorder(ASD), n (%)			NA			<0.001			0.31			0.98

No	NA	NA		7218 (89.5)	7032 (87.2)		402 (51.5)	423 (54.2)		5528 (85.2)	5530 (85.2)	
Yes	NA	NA		845 (10.5)	1031 (12.8)		378 (48.5)	357 (45.8)		964 (14.8)	962 (14.8)	
Attention Deficit/Hyperactivity Disorder(ADHD), n (%)			0.41			NA			0.168			0.012
No	1388 (55.3)	1418 (56.5)		NA	NA		428 (54.9)	456 (58.5)		4957 (76.4)	5078 (78.2)	
Yes	1122 (44.7)	1092 (43.5)		NA	NA		352 (45.1)	324 (41.5)		1535 (23.6)	1414 (21.8)	
Intellectual Disability			<0.001			0.026			NA			0.001
No	2262 (90.1)	2182 (86.9)		7805 (96.8)	7752 (96.1)		NA	NA		6319 (97.3)	6252 (96.3)	
Yes	248 (9.9)	328 (13.1)		258 (3.2)	311 (3.9)		NA	NA		173 (2.7)	240 (3.7)	
Speech or language disorder, n (%)			0.182			0.078			0.673			NA
No	1107 (44.1)	1155 (46.0)		6571 (81.5)	6482 (80.4)		175 (22.4)	183 (23.5)		NA	NA	
Yes	1403 (55.9)	1355 (54.0)		1492 (18.5)	1581 (19.6)		605 (77.6)	597 (76.5)		NA	NA	
Congenital Heart Condition, n (%)			0.444			0.003			0.012			<0.001
No	2374 (94.6)	2387 (95.1)		7826 (97.1)	7757 (96.2)		717 (91.9)	686 (87.9)		6296 (97.0)	6177 (95.1)	
Yes	136 (5.4)	123 (4.9)		237 (2.9)	306 (3.8)		63 (8.1)	94 (12.1)		196 (3.0)	315 (4.9)	
Heart Condition Currently, n (%)			0.698			0.002			0.033			<0.001
Does not have condition	2365 (94.2)	2375 (94.6)		7803 (96.8)	7718 (95.7)		711 (91.2)	680 (87.2)		6273 (96.6)	6153 (94.8)	
Ever, but not currently	82 (3.3)	81 (3.2)		152 (1.9)	196 (2.4)		42 (5.4)	66 (8.5)		126 (1.9)	188 (2.9)	
Currently has condition	63 (2.5)	54 (2.2)		108 (1.3)	149 (1.8)		27 (3.5)	34 (4.4)		93 (1.4)	151 (2.3)	
Severity of Current Heart Condition, n (%)			<0.001			<0.001			0.033			0.001
Does not currently have condition	2428 (96.7)	2429 (96.8)		7911 (98.1)	7867 (97.6)		738 (94.6)	714 (91.5)		6366 (98.1)	6304 (97.1)	
Mild	59 (2.4)	58 (2.3)		113 (1.4)	145 (1.8)		30 (3.8)	42 (5.4)		99 (1.5)	136 (2.1)	
Moderate	18 (0.7)	19 (0.8)		26 (0.3)	44 (0.5)		7 (0.9)	20 (2.6)		23 (0.4)	36 (0.6)	
Severe	5 (0.2)	4 (0.2)		13 (0.2)	7 (0.1)		5 (0.6)	4 (0.5)		4 (0.1)	16 (0.2)	

Abbreviations: IQR, interquartile range; NA, not applicable.

Supplementary Table 5. Association between genetically predicted congenital heart disease and neurodevelopmental disorders using inverse-variance weighted, Weighted median and MR-Egger methods.

Outcome	Number of SNPs	IVW		Weighted median		MR-Egger estimate	
		OR(95%CI)	p value	OR(95%CI)	p value	OR(95%CI)	p value
Autism Spectrum Disorder	10	1.04(0.97 - 1.12)	0.2523	1.03(0.93 - 1.13)	0.5705	0.96(0.84 - 1.11)	0.6172
Attention deficit/hyperactivity disorder	9	0.98(0.91 - 1.06)	0.5891	1.02(0.9176523 - 1.126230)	0.7526	0.86(0.70 - 1.04)	0.1673
Childhood intelligence	4	0.92(0.83 - 1.03)	0.1512	0.96(0.84 - 1.08)	0.4758	0.71(0.41 - 1.21)	0.3303
Speech and linguistic disorders	14	1.01(0.87 - 1.16)	0.9396	1.03(0.85 - 1.25)	0.7424	0.73(0.51 - 1.03)	0.0957

Abbreviations: SNP, single nucleotide polymorphism; OR, odds ratio; CI, confidence interval; IVW, inverse-variance-weighted (random-effects model); MR, Mendelian randomization.

Supplementary Table 6. Heterogeneity and sensitivity analyses in Mendelian randomization study using Cochran Q test, MR-Egger and MR-PRESSO methods.

Outcome	Number of SNPs	Cochran's Q statistic	p value for Cochran Q test	p value for MR-Egger intercept	p value for MR-PRESSO global test
Autism Spectrum Disorder	10	9.8916	0.3593	0.2268	0.367
Attention deficit/hyperactivity disorder	9	6.2199	0.6226	0.1920	0.641
Childhood intelligence	4	3.3118	0.3460	0.4216	0.409
Speech and linguistic disorders	14	8.1261	0.8353	0.0662	0.857

Abbreviations: SNP, single nucleotide polymorphism; MR, Mendelian randomization; PRESSO, Pleiotropy Residual Sum and Outlier.

Supplementary Figure 1. The scatter plot and results of leave-one-out analysis for autism spectrum disorder.

A. The scatter plot

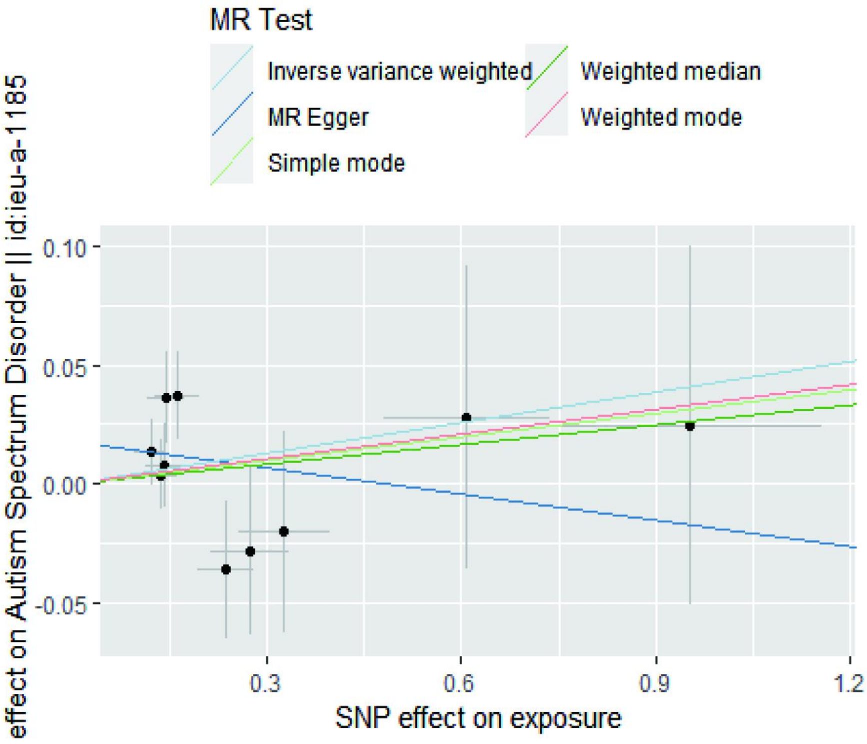

B. Result of leave-one-out analysis

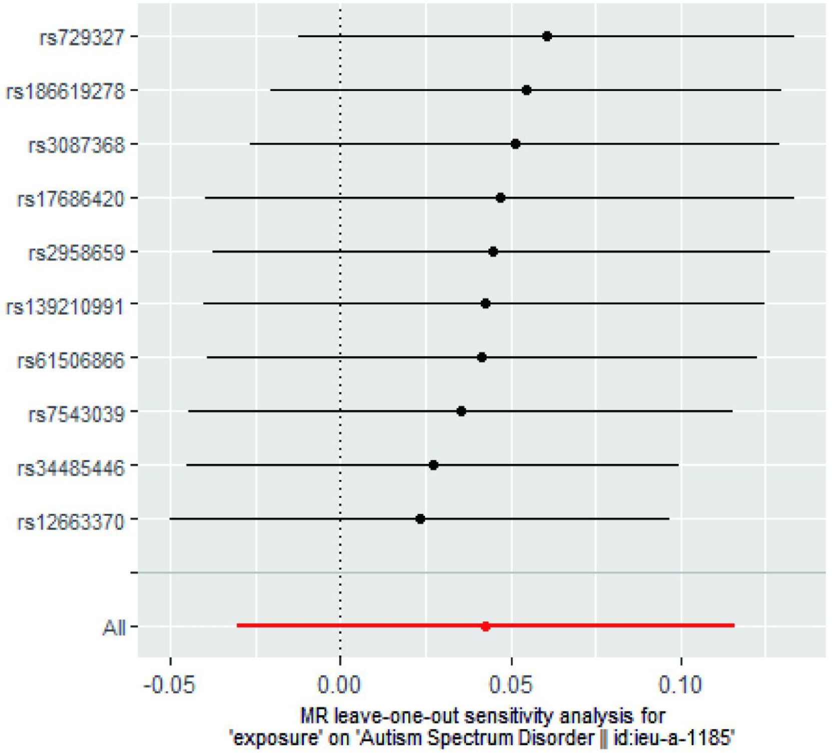

Supplementary Figure 2. The scatter plot and results of leave-one-out analysis for attention-deficit/hyperactivity disorder.

A. The scatter plot

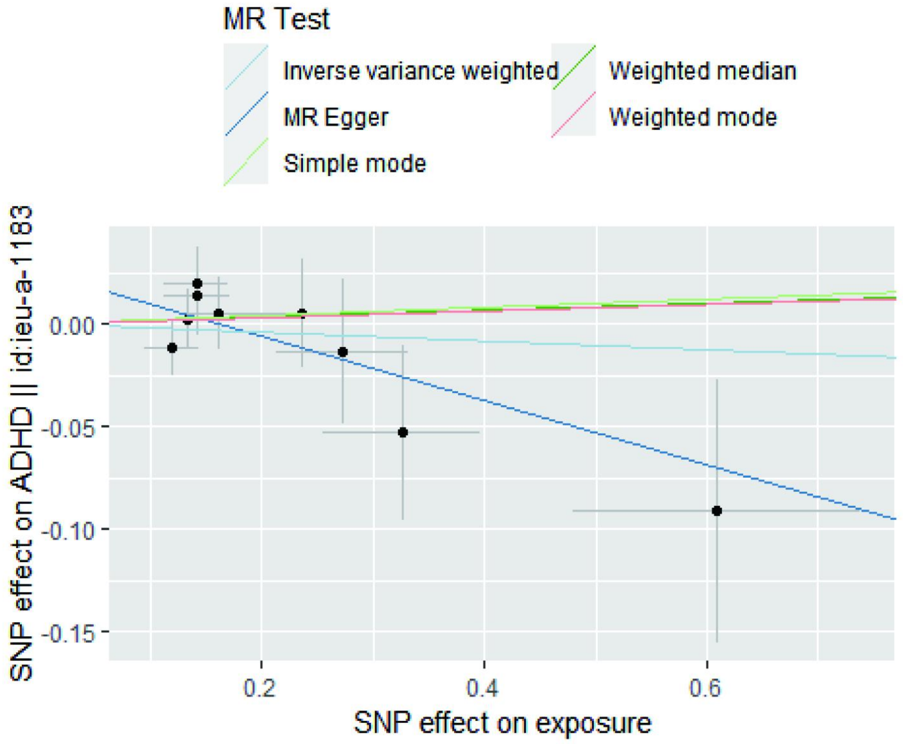

B. Result of leave-one-out analysis

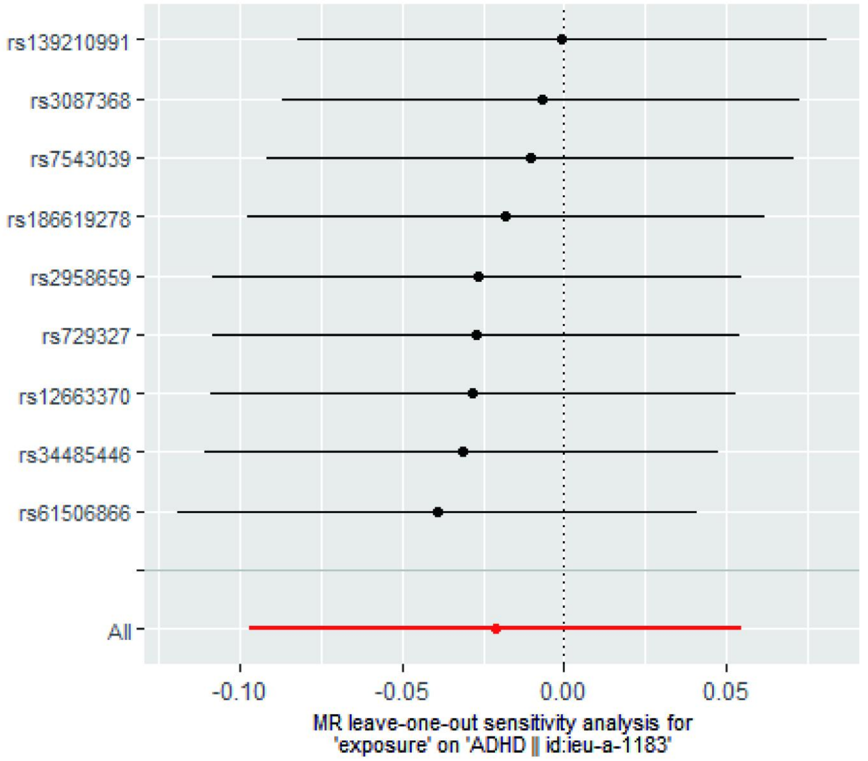

Supplementary Figure 3. The scatter plot and results of leave-one-out analysis for intellectual disability.

A. The scatter plot

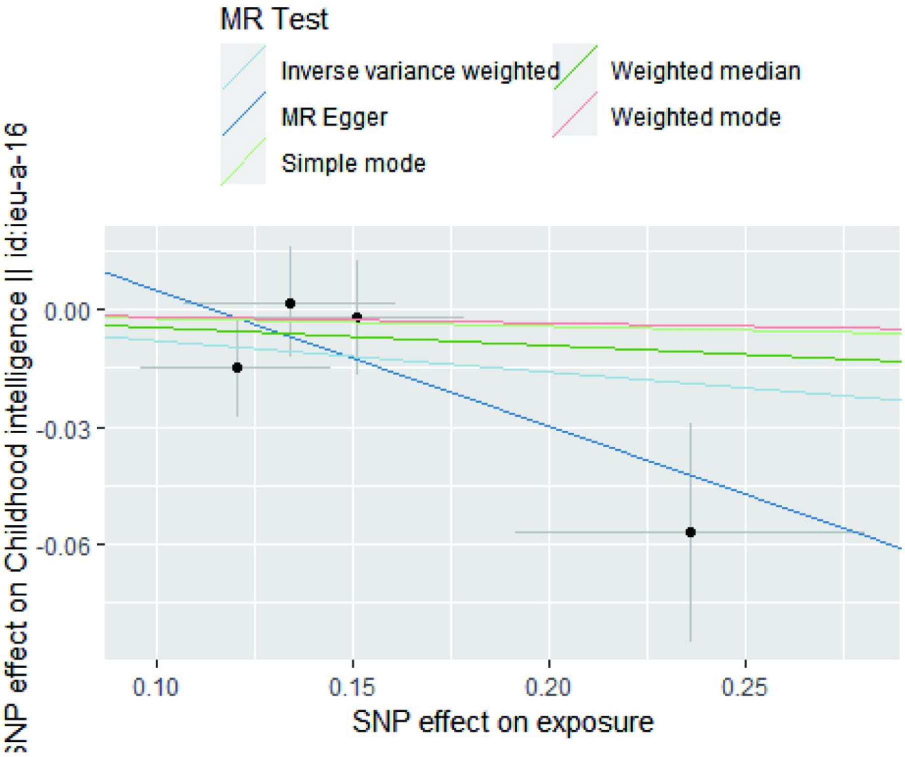

B. Result of leave-one-out analysis

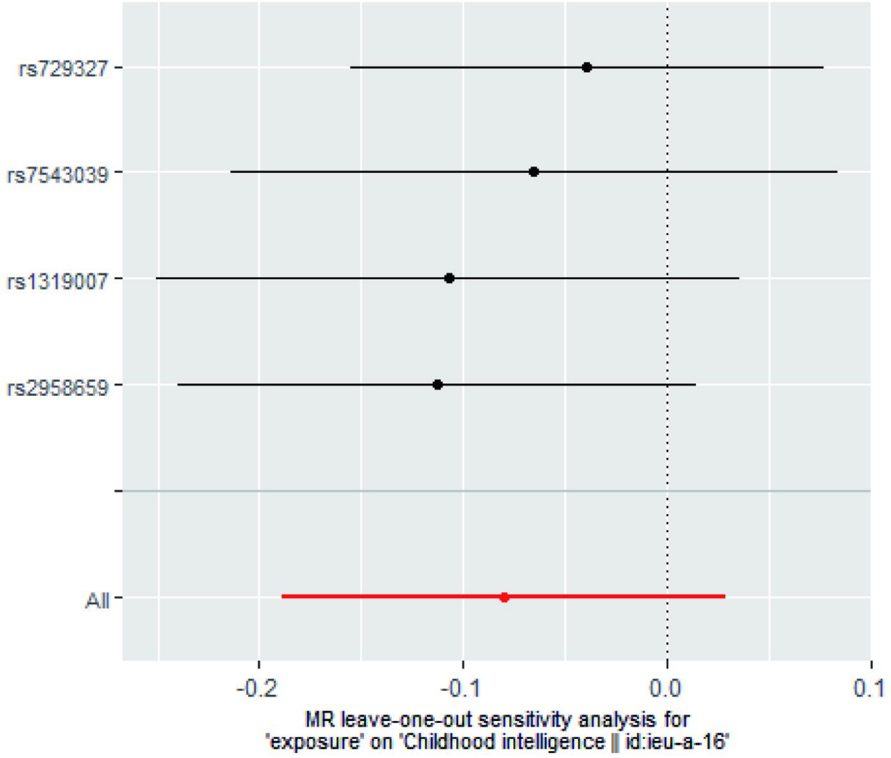

Supplementary Figure 4. The scatter plot and results of leave-one-out analysis for speech or language disorder.

A. The scatter plot

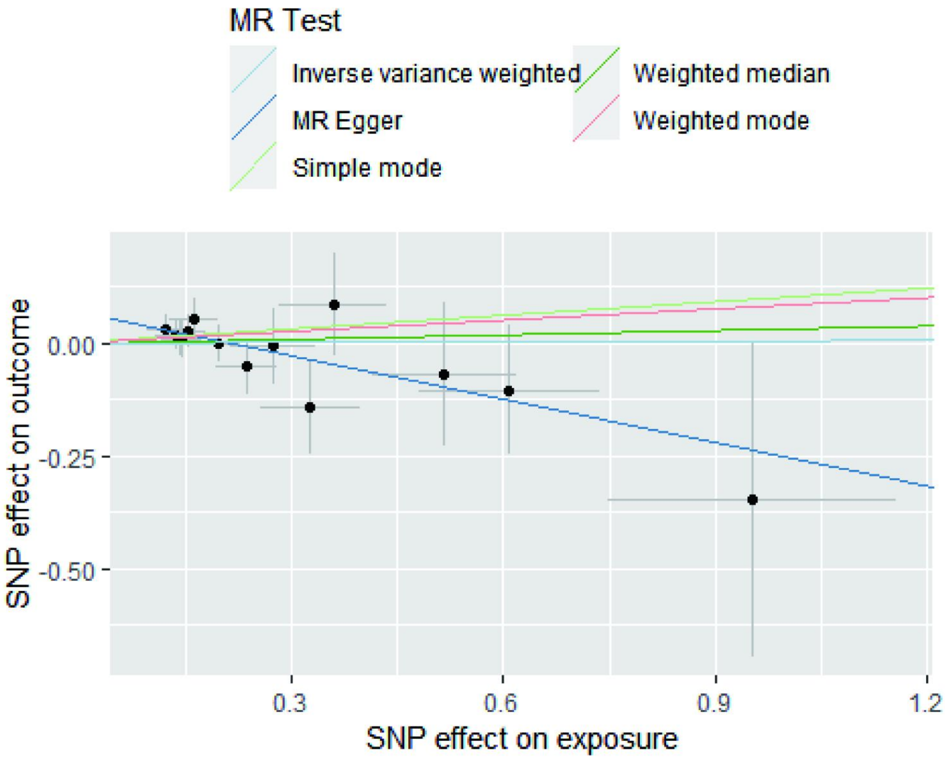

B. Result of leave-one-out analysis

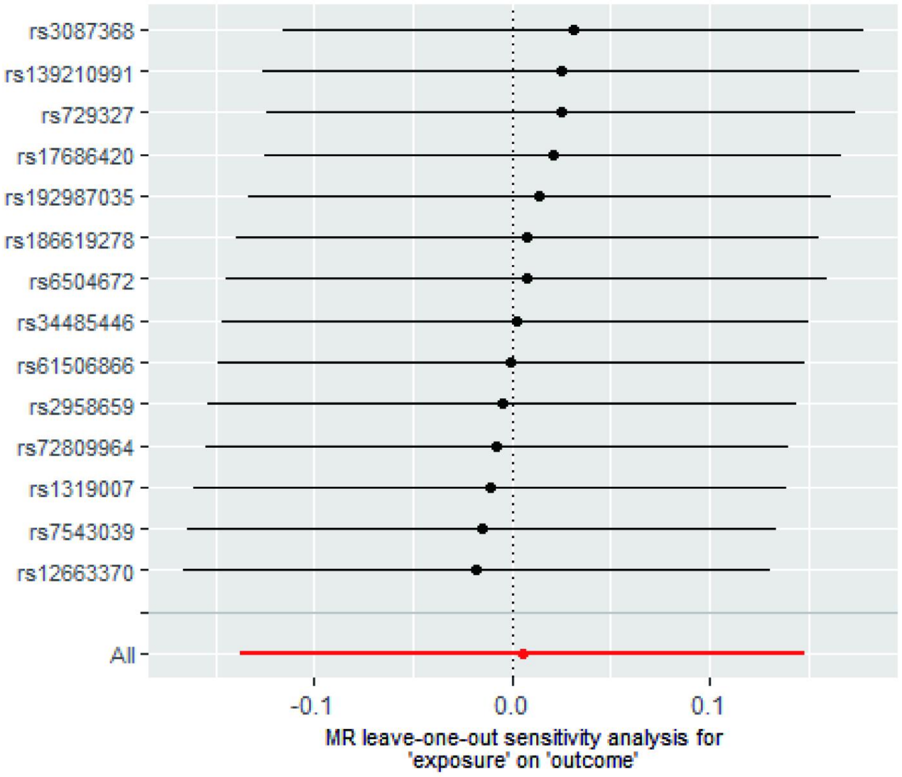

Supplement: Supplementary file 1 — Supplementary Material 1 [file 13052_2024_1610_MOESM1_ESM.pdf]
